# Supplementary material for: Unliganded Progesterone Receptor Governs Estrogen Receptor Gene Expression by Regulating DNA Methylation in Breast Cancer Cells
Source: Cancers (Basel). 2018 Oct 5;10(10):371. doi: 10.3390/cancers10100371 (PMC6210734; doi:10.3390/cancers10100371)
Supplement: Supplementary file 1 [file cancers-10-00371-s001.pdf]

Unliganded Progesterone Receptor Governs Estrogen Receptor Gene Expression by Regulating DNA Methylation in Breast Cancer Cells

Gaetano Verde, Lara I. De Llobet, Roni H.G. Wright, Javier Quilez, Sandra Peiró, François Le Dily and Miguel Beato

Supplementary Material

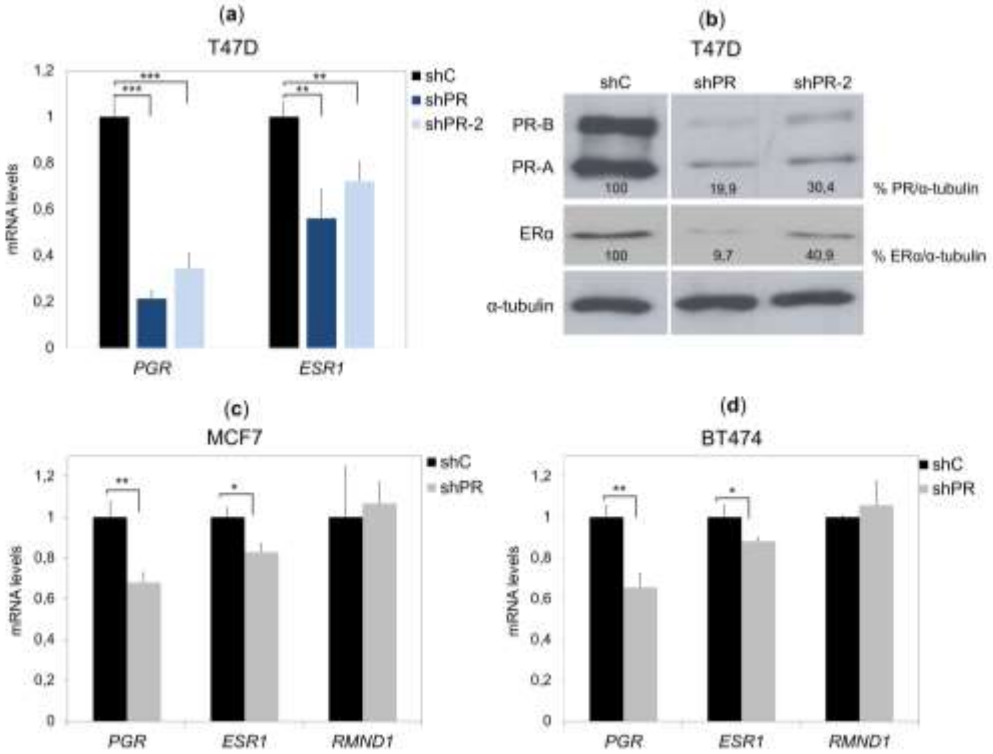

Figure S1. Depletion of PR reduces the *ESR1* expression in hormone-fre breast cancer cells. (**a**) Gene-specific mRNA expression measured by quantitative RT-PCR in T47D cells transduced with shRNA against PR (shPR, clone trcn0000010776; shPR-2, clone trcn0000003321) or scrambled shRNA (shC). The gene-specific expression levels were normalized to *GAPDH* expression and are represented as relative values in the T47D cells. *PGR*, PR-encoding gene; *ESR1*, ER-encoding gene. Error bars represent the standard deviation (SD) of three independent experiments. ** $p \leq 0.01$, *** $p \leq 0.005$, unpaired two-tailed Student's *t*-test. (**b**) PR and ERα protein levels measured by Western blot in T47D cells transduced with shRNA against PR (shPR, clone trcn0000010776; shPR-2, clone trcn0000003321) or scramble shRNA (shC). α-tubulin protein was used as loading control. The vertical white line depicts a removed lane between the two samples. (**c** and **d**) Gene-specific mRNA expression measured by quantitative RT-PCR in MCF7 cells (**c**) and BT474 (**d**) transduced with shRNA against PR (shPR, clone trcn0000010776) or scrambled shRNA (shC). The gene-specific expression levels were normalized to *GAPDH* expression and are represented as relative values in the MCF7 (**c**) or BT474 (**d**) cells. Error bars represent the SD of three independent experiments. * $p \leq 0.05$, ** $p \leq 0.01$, unpaired two-tailed Student's *t*-test.

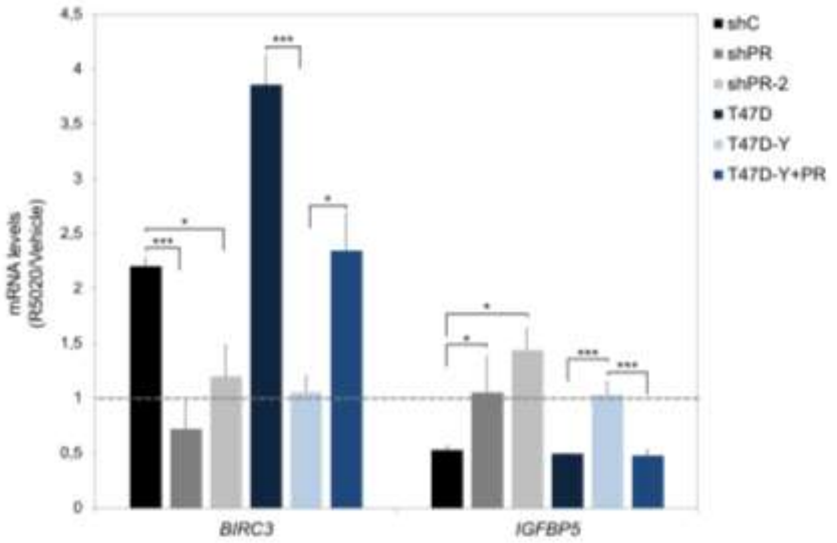

Figure S2. PR depletion and PR-rescue affect progestin-mediated gene transcription. Short hairpin control (shC), PR-depleted cells (shPR-1, clone trcn0000010776 and shPR-2, clone trcn0000003321), T47D cells, PR-deficient (T47D-Y+PR) cells and PR-rescue cells (T47D-Y+PR) were treated with progestin (R5020) or ethanol (vehicle) for 6 hours, at which point mRNA expression of PR-induced genes (*BIRC3*) and a PR-repressed gene (*IGFBP5*) was measured by quantitative RT-PCR. The gene expression was normalized to *GAPDH* expression and is represented as fold change relative to the vehicle (R5020/vechicle). Error bars represent the SD of three independent experiments. * $p \leq 0.05$, *** $p \leq 0.005$, unpaired two-tailed Student's *t*-test.

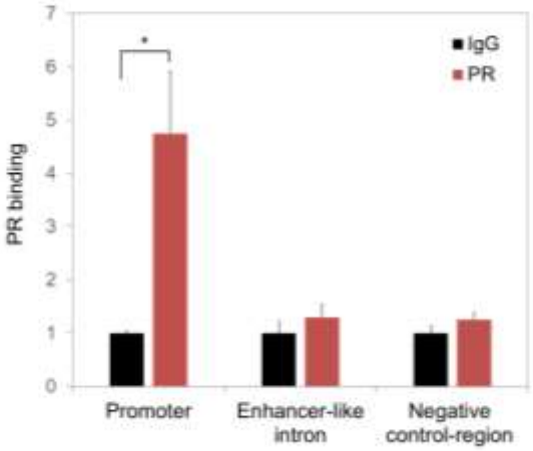

Figure S3. PR binds at *ESR1* gene promoter in MCF7 breast cancer cells. ChIP assay was performed with a specific antibody against PR or total rabbit IgG. Specific binding was assessed by qPCR amplification of the *ESR1* gene promoter, an enhancer-like intronic sequence, and a genomic region localized at 3′-end of the enhancer-like intron (negative control region). PR binding is represented as fold change relative to the negative control (IgG). Error bars represent the SD of three independent experiments. * $p \leq 0.05$, unpaired two-tailed Student's *t*-test.

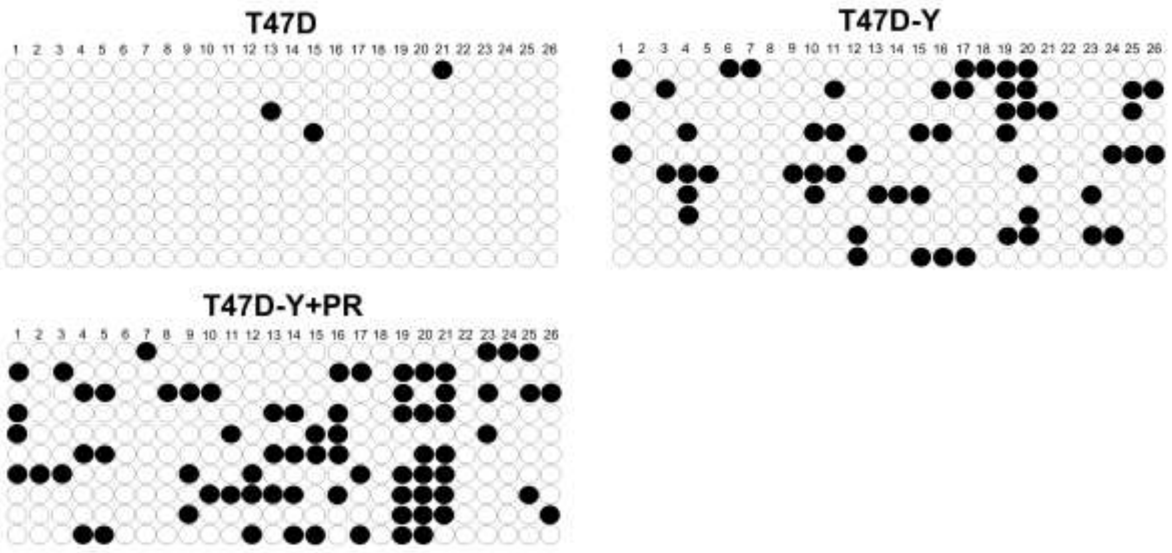

Figure S4. The loss of PR increases DNA methylation at *ESR1* promoter. DNA methylation analysis of *ESR1* promoter determined by direct bisulfite sequencing in T47D control cells, PR-deficient cells (T47D-Y) and PR-rescued cells (T47D-Y+PR). Each line corresponds to a single template DNA molecule cloned. The genomic region analyzed contains 26 CpG and filled circles designate the methylated cytosines, while opened circles designate unmethylated cytosines.

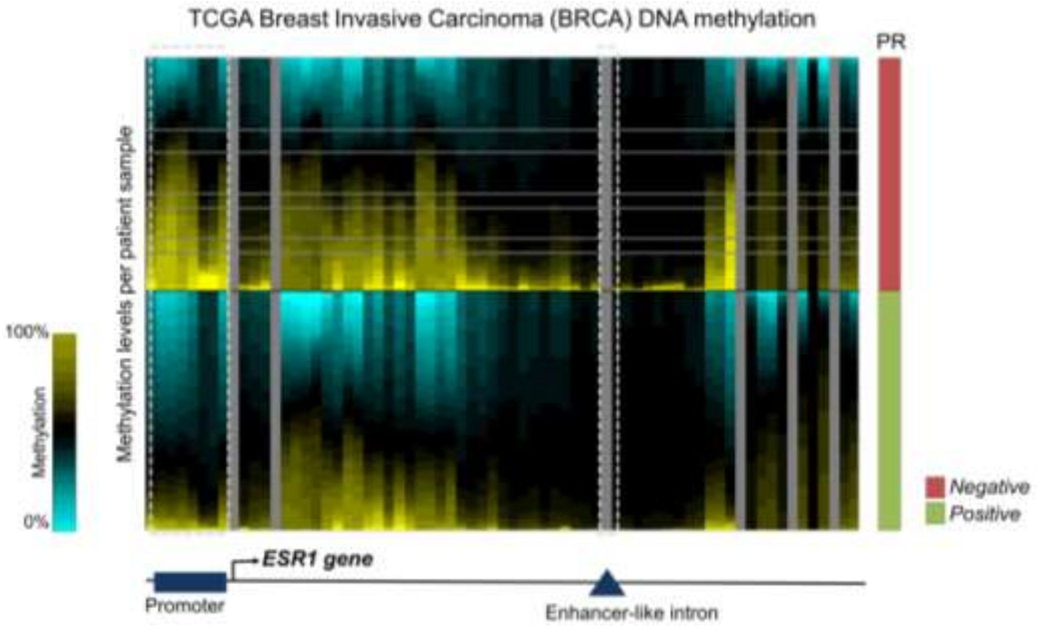

Figure S5. DNA methylation at the *ESR1* locus is higher in PR-negative than PR-positive breast invasive cancers. The TCGA database was used to assess the percentage of methylation across the *ESR1* gene in breast invasive carcinoma (BRCA) dataset, including both ER+ and ER- cancers (n = 837); DNA methylation levels had been assayed previously using human methylation 450 arrays and the *ESR1* percentage methylation levels across all patient samples were plotted and stratified based on PR status (right). The vertical gray lines represent the intronic regions. The areas corresponding to the gene promoter and the enhancer-like intronic region are highlighted with a dashed box.

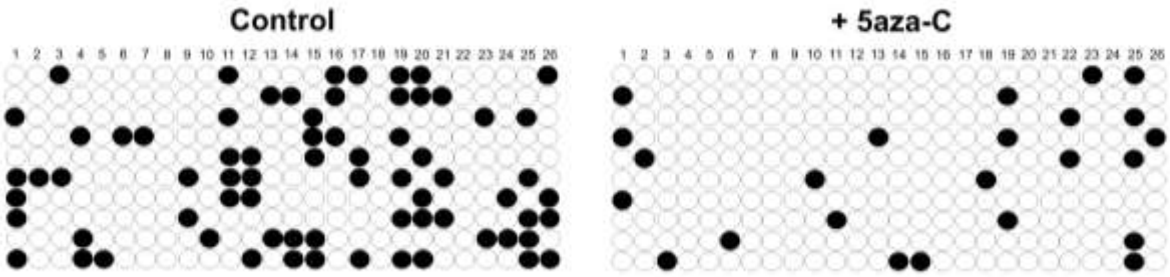

Figure S6. The 5-azacytidine (5aza-C) demethylates the *ESR1* promoter. DNA methylation analysis of *ESR1* promoter determined by direct bisulfite sequencing in PR-rescued cells (T47D-Y+PR) treated with 5-azaC or vehicle (control). Each line corresponds to a single template DNA molecule cloned; The genomic region analyzed contains 26 CpG and filled circles designate the methylated cytosines, while opened circles designate unmethylated cytosines.

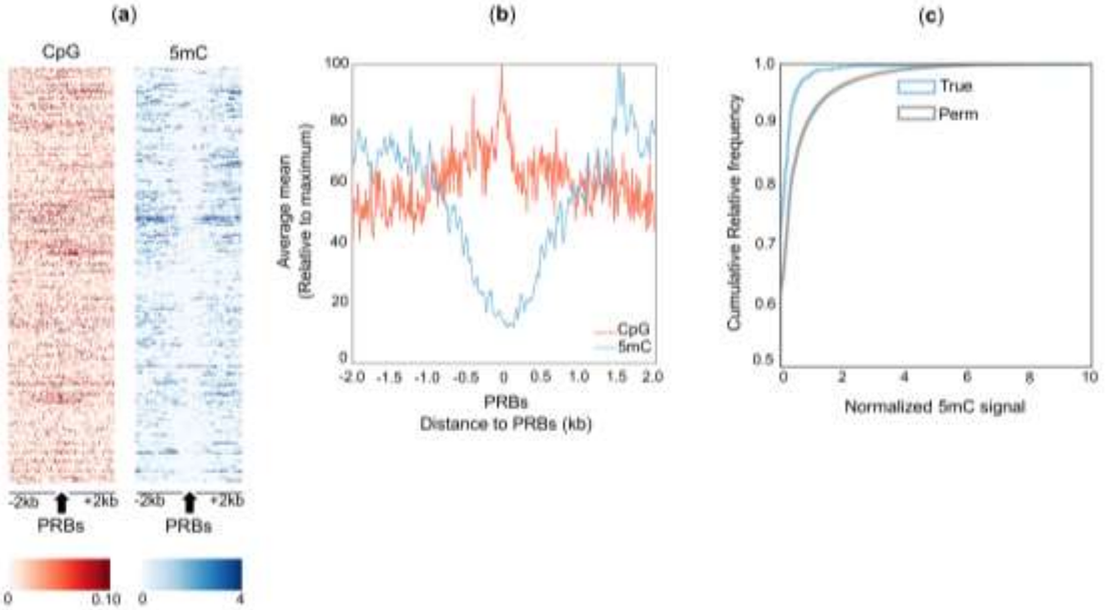

Figure S7. The PR binding sites are low methylated in hormone-deprived breast cancer cells. (**a**) Heatmaps of the normalized CpG content and DNA methylation (5mC) analyzed by MeDIP-seq around all PR binding sites (476 PRBs) in hormone-deprived T47D cells. (**b**) Average signal intensity of the normalized CpG content and DNA methylation (5mC) signal centered around all PRBs (476) in hormone-deprived T47D cells. DNA methylation was significantly lower at PRB sites than their flanking region (−/+ 2000 bp). (Wilcoxon test: $p = 5.07 \times 10^{-16}$). (**c**) Normalized DNA methylation (5mC) signal in PRB sites (True) or in random genomic regions of the same size as PRBs (Perm; data points were pooled from 1,000 permutations). DNA methylation was significantly lower at PRB sites than at random genomic regions (Mann-Whitney U test: $p = 3.85 \times 10^{-9}$).

Table S1. Oligonucleotide sequences.

**Primers RT-qPCR**	**Sequence: 5′–3′**
ESR1 forward	TCTATTCCGAGTATGATCCTACCA
ESR1 reverse	CAGACGAGACCAATCATCAG
PR forward	GGCGAGAGGCAACTTCTTTC
PR reverse	CATCTGCCCACTGACGTGTT
RMND1 forward	ATCTTGAGACAGATTTCCCAG
RMND1 reverse	ACCTTCCAACCATGAAATCC
pS2 forward	TTGTGGTTTTCCTGGTGTCA
pS2 reverse	CCGAGCTCTGGGACTAATCA
GAPDH forward	TTGATTTTGGAGGGATCTCG
GAPDH reverse	GAGTCAACGGATTTGGTCGT
BIRC3 forward	GACAGCCCAGGAGATGAAAA
BIRC3 reverse	CACGGCAGCATTAATCACAG
CD44 forward	AGAAGGTGTGGGCAGAAGAAA
CD44 reverse	CATTCTGCAGGTTCCTTGTCT
IGFBP5 forward	GAGCTGAAGGCTGAAGCAGT
IGFBP5 reverse	GAATCCTTTGCGGTCACAAT
**Primers bisulphite-sequencing**	**Sequence: 5′–3′**
ESR1 promoter forward 1st round	TTTATATTAAAGTATTTGGGATGGTTTT
ESR1 promoter forward 2nd round	GATGGTTTTATTGTATTAGATTTAAGGG
ESR1 promoter reverse	CTATTAAATAAAAAAAAACCCCCCAAA
**Primers ChIP-qPCR and MeDIP-qPCR**	**Sequence: 5′–3′**
Promoter forward	CAGCAGCGACGACAAGTAAA
Promoter reverse	ACTGGTCTCCCGAGCTCATA
Enhancer-like intron forward	GGAAAGCCGGCAGTTACA
Enhancer-like intron reverse	GGAGGCATGAAAGCCCTAC
Negative control region forward	CATGGACTGGCAGAGCATAA
Negative control region reverse	CCCAACATATTGAAGGTGTGC
**Oligonucleotides EMSA**	**Sequence: 5′–3′**
No CpG probe forward	GTTGAGTCAGAACAGTTTGTTCTCTAGGCAC
No CpG probe reverse	GTGCCTAGAGAACAAACTGTTCTGACTCAAC
One CpG probe forward	GTTGAGTCAGAACGGTTTGTTCTCTAGGCAC
One CpG probe reverse Unmethylated	GTGCCTAGAGAACAAACCGTTCTGACTCAAC
One CpG probe reverse Methylated	GTGCCTAGAGAACAAACC*GTTCTGACTCAAC
Two CpGs probe forward	GTTGAGTCAGAACGGTTCGTTCTCTAGGCAC
Two CpGs probe reverse Unmethylated	GTGCCTAGAGAACGAACCGTTCTGACTCAAC
Two CpGs probe reverse Methylated	GTGCCTAGAGAAC*GAACC*GTTCTGACTCAAC

* Methylated cytosine.
